# DISABILITY, COMMUNITY PARTICIPATION, AND LIFE SATISFACTION IN WORKING-AGE CANCER SURVIVORS

**DOI:** 10.2340/jrm.v58.46071

**Published:** 2026-10-29

**Authors:** Ada CHEN, Jing TAO, Lidian CHEN

**Affiliations:** 1 Kessler Foundation, East Hanover, NJ, USA; 2 Fujian University of Traditional Chinese Medicine, Fuzhou, China

**Keywords:** cancer survivors, International Classification of Functioning, Disability and Health, social participation, personal satisfaction, social support

## Abstract

**Objective:**

To examine whether community participation mediates the disability–life satisfaction association in working-age cancer survivors, and whether contextual factors moderate this pathway.

**Design:**

Cross-sectional online survey.

**Subjects:**

2,999 working-age US cancer survivors, employed or seeking employment (mean age 42.8 years; 53.1% male; 75.7% in remission).

**Methods:**

Disability (World Health Organization Disability Assessment Schedule 2.0), perceived community participation (5-item Wisconsin Community Participation Scale), and life satisfaction (Satisfaction With Life Scale) were assessed. A single-mediator model was estimated with bias-corrected bootstrap; 7 moderators were tested with false-discovery-rate correction.

**Results:**

Disability was associated with lower participation (β = −0.14) and life satisfaction (β = −0.12); participation with greater life satisfaction (β = 0.57). The indirect association (β = −0.08, 95% CI −0.10 to −0.06) accounted for 39.8% of the modest total association, but for only 13.0% when the full 12-item participation scale was used. At high instrumental support the conditional indirect association was indistinguishable from zero; at high self-efficacy and hope it reversed in sign.

**Conclusion:**

In this cross-sectional study, community participation statistically accounted for a measure-dependent part of the disability–life satisfaction association, and contextual factors were associated with differential attenuation across pathway segments. These hypothesis-generating findings require longitudinal confirmation before informing practice.

## LAY ABSTRACT

Cancer survivors often live with lasting health problems that limit their daily functioning. This study asked whether such limitations reduce life satisfaction partly because they reduce participation in everyday community life, such as working, exercising, and socializing. In a survey of 2,999 cancer survivors in the United States, up to approximately 40% of the link between disability and lower life satisfaction was explained by reduced community participation. Survivors were not all affected in the same way: those with strong practical support from family and friends showed little of this pattern, and those with high confidence and hope appeared to maintain their well-being despite disability. Because everyone was surveyed at a single point in time, these patterns need to be confirmed in studies that follow people over time. Still, the findings suggest that helping survivors stay engaged in their communities may be as important for life satisfaction as treating physical symptoms.

The US cancer survivor population now exceeds 18 million and is projected to surpass 22 million by 2030 (1). National surveillance data indicate that 27.9% of US cancer survivors report mobility disability and 7.4% report self-care disability (2), and an estimated 40% experience long-term physical or cognitive consequences of cancer or its treatment that constrain valued activities (3). The question that follows is mechanistic: through which processes is post-treatment disability linked to lower life satisfaction, and which contextual resources attenuate those links.

The International Classification of Functioning, Disability and Health (ICF) (4) conceptualizes functioning and disability as the dynamic interaction of body functions and structures, activities, and participation, influenced by health conditions and by environmental and personal contextual factors. Within this framework, participation (involvement in life situations) is both a downstream outcome of impairment and an upstream determinant of how disability affects quality of life, and contextual factors are specified as moderators of the relationships among ICF components. Importantly, the ICF is a multidimensional biopsychosocial framework rather than a linear causal model; the mediation pathway examined here therefore represents a single testable hypothesis derived from the framework, not a comprehensive test of the framework itself.

Two empirically tractable predictions follow. First, participation should mediate, rather than merely accompany, the relationship between disability and subjective well-being. Second, contextual factors should attenuate this mediated pathway, with the locus of attenuation differing by factor and by pathway segment. Direct evidence for either prediction remains limited: activity limitations fully mediated the impairment to participation-restriction relationship in osteoarthritis (5), and community participation both mediated and moderated the disability to life-satisfaction relationship in fibromyalgia (6), but neither study examined cancer survivors or tested moderation separately at each pathway segment.

Survivorship programmes commonly assume that contextual resources such as social support, exercise, and psychological resilience improve outcomes additively. If these resources instead buffer specific segments of the disability to well-being pathway, they are not clinically interchangeable: mobilizing instrumental support to remove a transportation barrier addresses a different point in the cascade than building hope.

The current study had 3 aims: to test whether community participation mediates the association between disability and life satisfaction in a national sample of working-age US cancer survivors; to test whether 7 contextual factors spanning the environmental, personal, and behavioural categories of the ICF’s contextual factors component moderate this pathway, and to identify the segment at which each factor acts; and to estimate, for each moderator surviving correction for multiple testing, the level at which the conditional indirect association of disability with life satisfaction was substantially attenuated or reduced to statistical non-significance. Because the design is cross-sectional, mediation and moderation are used throughout in their statistical sense, and all estimates are interpreted as conditional associations rather than causal effects.

## Methods

### Design and reporting

This cross-sectional online survey was reported in line with the STROBE statement for observational studies.

### Participants

Data were drawn from *Cancer Survivors in the Workforce: A Comprehensive National Study*, conducted at the Kessler Foundation. The study protocol was reviewed and approved by the Kessler Foundation Institutional Review Board (protocol no. E-1299-25), and all participants provided informed consent electronically before beginning the survey. Adults aged ≥ 18 years residing in the United States, with a self-reported physician-confirmed cancer diagnosis, who were currently employed or actively seeking employment, were recruited via CloudResearch Managed Research, a vetted academic research participant platform that has been validated against probability-based national samples for health and behavioural research (15–17). The survey was hosted on Qualtrics; data were collected between September and October 2025. Of 3,852 responses initially exported (including 576 incomplete submissions and 2 metadata rows), 224 were removed at screening (the 2 metadata rows and 222 responses failing 2 or more of 5 embedded attention checks), yielding 3,628 retained records (3,062 completed and 566 incomplete submissions). Listwise deletion across all model variables produced a final analytic sample of 2,999. The 629 records removed comprised 566 incomplete submissions, in which respondents had exited the survey before the sections containing the model variables, and 63 completed surveys with item non-response on at least 1 model variable (most often current employment, *n* = 36; regular exercise, *n* = 34; or WHODAS 2.0 items, *n* = 27); no imputation was performed.

### Measures

*Disability (limitations in functioning; ICF activity and participation components).* The 12-item World Health Organization Disability Assessment Schedule 2.0 (WHODAS 2.0) (18) was scored as the item mean (5-point scale, 1 = none to 5 = extreme or cannot do; higher scores indicate greater disability; α = 0.95). The WHODAS 2.0 summary score is referred to throughout as disability, in the sense of limitations in functioning; the instrument assesses activity limitations and participation-related difficulties rather than body functions or structures. Two WHODAS 2.0 items belong to its participation domain (joining community activities; emotional impact of health problems); overlap with the participation mediator was addressed in a sensitivity analysis excluding them.

*Perceived community participation (ICF participation).* A 5-item subset of the Wisconsin Community Participation Scale (WCPS) (19) was used, covering full-time work, leisure and recreational activities, independent living, social, and physical activity. Items were rated on a 5-point scale comparing actual with desired participation; the mean score was used (α = 0.82). Because responses index satisfaction with one’s level of participation relative to personal standards rather than enacted frequency of participation, the construct is referred to as perceived community participation (hereafter, participation). The subset was selected from the 12-item scale by an a priori item analysis that removed role-specific items (e.g., parenting, spousal, and student roles) inapplicable to many respondents (Appendix S1); a sensitivity analysis using the full scale is reported in the Results section.

*Life satisfaction*. The 5-item Satisfaction With Life Scal (SWLS) (20) was summed across items rated on a 7-point agreement scale (range 5–35; α = 0.92).

*Moderators* (ICF contextual factors). Moderators were grouped into 3 categories (the ICF distinguishes but does not formally classify personal factors). *Environmental*: cancer rehabilitation receipt (yes/no: occupational therapy, physical therapy, or related rehabilitation received since diagnosis) and the 4-item Patient-Reported Outcomes Measurement Information System (PROMIS) Instrumental Support short form (α = 0.92) (21). *Personal*: a 5-item social self-efficacy measure assessing confidence in social interactions on a 5-point scale (α = 0.89; social rather than generalized self-efficacy); the 8-item Adult Trait Hope Scale (9) (α = 0.93); and the 12-item Core Self-Evaluations Scale (12) (α = 0.89). *Behavioural*: regular exercise at least once per week (yes/no, single item) and current employment (yes/no, single item).

*Covariates.* These were age (continuous), gender (1 = female), educational attainment (continuous, 1–6), cancer rehabilitation receipt, and remission status. An extended, cancer-specific covariate set was examined in sensitivity analysis: time since first diagnosis (years), recurrence (yes/no), second primary cancer (yes/no), known metastatic disease (yes/no), number of treatment modalities received (surgery, chemotherapy, hormone therapy, targeted therapy, immunotherapy, radiation; range 0–6), pain intensity, and fatigue. Cancer stage and current treatment status were not collected and could not be adjusted for.

### Statistical analysis

Analyses used R 4.3.3 (R Foundation for Statistical Computing, Vienna, Austria) and the *lavaan* package, version 0.6-17 (22). Continuous variables were standardized; binary variables were entered as observed 0/1 indicators. Models were estimated with maximum likelihood; missing data were handled by listwise deletion (see Participants). The mediation models are saturated, so global fit indices are not applicable. Bias-corrected bootstrap confidence intervals were used because they do not assume a normal sampling distribution for the indirect term; linearity and homoscedasticity were checked by inspection of residual plots.

The base single-mediator model specified disability → participation → life satisfaction. Path *a* was the disability-to-participation coefficient; path *b* was the participation-to-life-satisfaction coefficient adjusted for disability; path *c′* was the direct disability-to-life-satisfaction coefficient adjusted for participation. The indirect association (*a* × *b*) was tested with bias-corrected bootstrap 95% confidence intervals (5,000 resamples).

For each of the 7 moderators, a moderated mediation (MM) model added 3 interaction terms (moderator × disability on participation; moderator × participation on life satisfaction; moderator × disability on life satisfaction). The Benjamini–Hochberg false discovery rate (FDR) correction was applied across the 21 interaction tests.

For each moderator with at least 1 significant interaction, conditional (simple) slopes for paths *a*, *b*, and *c′*, the conditional indirect association (*a* × *b*), and the conditional total association (*c′* + *a* × *b*) were computed at low (−1 SD or moderator = 0) and high (+1 SD or moderator = 1) moderator levels, with bias-corrected bootstrapped 95% CIs (5,000 resamples). Buffering denotes an interaction that weakens an adverse association (a positive interaction on path *a* or *c′*); because path *b* is positive, a negative path-*b* interaction, which indicates that life satisfaction was less closely associated with participation at higher moderator levels, is described as attenuation rather than buffering. Because conditional estimates were computed for multiple moderators, they are interpreted descriptively rather than as confirmatory tests.

Four sensitivity analyses assessed robustness of the base mediation: (*i*) a covariate-adjusted model (age, gender, education, cancer rehabilitation, remission status); (*ii*) an extended model adding the cancer-specific covariates described earlier; (*iii*) substitution of the full 12-item WCPS for the 5-item participation subset; and (*iv*) re-estimation with a 10-item WHODAS 2.0 score excluding the 2 participation-domain items.

## Results

### Sample characteristics

The analytic sample (*N* = 2,999) had a mean age of 42.8 (SD 12.1) years; 53.1% identified as male and 70.8% as White. Most were currently employed (90.2%) and in remission (75.7%). The most common cancer types were breast (19.7%), prostate (16.2%), and cervical (10.6%). Cancer rehabilitation had been received by 41.1%, regular exercise by 65.9%, and at least 1 functional impairment was reported by 79.1% (Table I).

**Table I:** Demographic and clinical characteristics of the analytic sample (*N* = 2,999)

Characteristic	*n* or mean	% or SD
Age (years), mean (SD)	42.8	12.1
Male	1,593	53.1%
White race	2,122	70.8%
Currently employed	2,705	90.2%
In remission	2,269	75.7%
Received cancer rehabilitation	1,233	41.1%
Regular exercise	1,976	65.9%
Any functional impairment	2,371	79.1%
Cancer type: breast	590	19.7%
Cancer type: prostate	486	16.2%
Cancer type: cervical	317	10.6%

Bivariate correlations among the core ICF variables were in the predicted directions: WHODAS correlated negatively with participation (*r* = −0.14) and life satisfaction (*r* = −0.20), and participation correlated positively with life satisfaction (*r* = 0.59; all *p* < 0.001) (Table II).

**Table II:** Pearson correlations among core ICF model variables (*N* = 2,999): all correlations significant at *p* < 0.001

Variable	1. WHODAS	2. Participation	3. SWLS
1. WHODAS (disability)	—		
2. Community participation	−0.14	—	
3. Life satisfaction (SWLS)	−0.20	0.59	—

### Base single-mediator model

Disability was associated with reduced participation (path *a*: β = −0.14, 95% CI −0.18 to −0.10) and reduced life satisfaction (path *c′*: β = −0.12, 95% CI −0.15 to −0.09). Participation was associated with greater life satisfaction adjusted for disability (path *b*: β = 0.57, 95% CI 0.54 to 0.60). The indirect association was significant (β = −0.08, 95% CI −0.10 to −0.06), accounting for 39.8% of the total association (β = −0.20). The model explained 36.1% of variance in life satisfaction and 2.0% of variance in participation (Fig. 1).

**Fig. 1:**
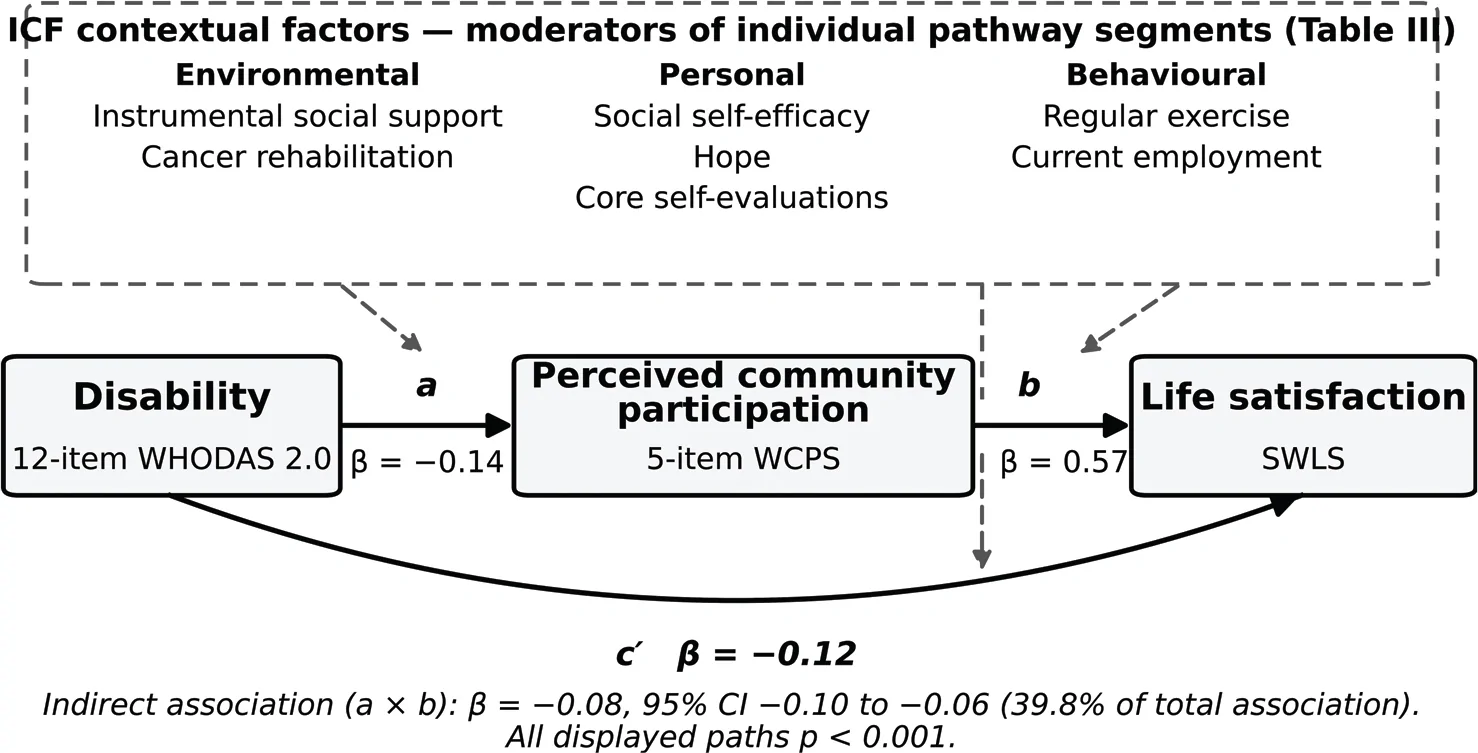
International Classification of Functioning, Disability and Health (ICF)-informed conceptual model with standardized path coefficients from the base single-mediator model (*N* = 2,999): disability (12-item World Health Organization Disability Assessment Schedule 2.0 [WHODAS 2.0]) → perceived community participation (5-item Wisconsin Community Participation Scale [WCPS]) → life satisfaction (Satisfaction With Life Scale [SWLS]). Solid arrows denote the mediation paths (a, b) and the direct path (c′); dashed arrows denote moderation of individual pathway segments by the 7 ICF contextual factors (segment-specific results in Table III). All displayed path coefficients significant at *p* < 0.001. The indirect association (β = −0.08, 95% CI −0.10 to −0.06) accounted for 39.8% of the total association.

### Moderated mediation

Across 21 interaction tests, 16 remained significant after FDR correction (Table III); the corresponding conditional slopes are given in Table IV. Path-*a* moderation was the most pervasive: for 6 of 7 moderators the interaction coefficient was positive and significant, so the negative disability–participation association was weaker at higher moderator levels. For the environmental and behavioural moderators this was a buffering pattern: the conditional path *a* weakened from β = −0.30 at low instrumental support to −0.01 at high support, from −0.24 without cancer rehabilitation to −0.10 with it, and from −0.22 without regular exercise to −0.06 with it. For the 3 personal resources the conditional slope changed sign, from −0.14 to +0.12 across levels of social self-efficacy, −0.11 to +0.12 across hope, and +0.01 to +0.24 across core self-evaluations: at high levels of these resources, greater disability was associated with greater perceived participation. Employment moderation of path *a* did not survive FDR correction (*p*FDR = 0.051). Path *b* (participation → life satisfaction) was moderated by core self-evaluations (interaction β = −0.12), hope (−0.08), exercise (−0.07), social self-efficacy (−0.04), and instrumental support (−0.03). Because path *b* is positive, these negative coefficients indicate attenuation rather than buffering: participation was less closely associated with life satisfaction at higher moderator levels (e.g., β = 0.48 at low vs 0.24 at high core self-evaluations; 0.44 vs 0.28 across levels of hope). Path *c′* (direct disability → life satisfaction) showed 2 opposite patterns. Cancer rehabilitation and instrumental support had positive interactions that weakened the negative direct association (from −0.18 to −0.10 and from −0.17 to −0.08), a buffering pattern. Social self-efficacy, hope, and core self-evaluations had negative interactions: conditional on each resource, the direct association was positive or near zero at low resource levels (β = +0.03, +0.08, and +0.20) and lower at high levels (−0.06, −0.02, and +0.07), that is, less favourable as the resource increased.

**Table III:** Standardized interaction coefficients for moderation of each pathway segment, with FDR-adjusted *p*-values

Moderator	Path a β	*p*_FDR_	Path b β	*p*_FDR_	Path c′ β	*p*_FDR_
Cancer rehabilitation	0.14*	0.002	0.02	0.538	0.07*	0.022
Instrumental support	0.15*	< 0.001	−0.03*	0.038	0.04*	0.010
Social self-efficacy	0.13*	< 0.001	−0.04*	0.007	−0.05*	0.002
Hope	0.12*	< 0.001	−0.08*	< 0.001	−0.05*	< 0.001
Core self-evaluations	0.11*	< 0.001	−0.12*	< 0.001	−0.07*	< 0.001
Exercise	0.15*	0.001	−0.07*	0.023	−0.01	0.868
Current employment	0.12	0.051	0.10	0.108	0.07	0.251

* *p*FDR < 0.05.

**Table IV:** Conditional (simple) slopes of each pathway segment and conditional indirect and total associations of disability with life satisfaction at low and high moderator levels, with bias-corrected bootstrapped 95% confidence intervals (CIs) (5,000 resamples)

Moderator and level	Path *a* β (95% CI)	Path *b* β (95% CI)	Path *c′* β (95% CI)	Indirect (*a* × *b*) β (95% CI)	Total (*c′* + *a* × *b*) β (95% CI)
Cancer rehabilitation Not received (low)	−0.24 (−0.29, −0.18)	0.55 (0.51, 0.58)	−0.18 (−0.22, −0.14)	−0.13 (−0.16, −0.10)	−0.31 (−0.36, −0.25)
Cancer rehabilitation Received (high)	−0.10 (−0.16, −0.03)	0.56 (0.52, 0.60)	−0.10 (−0.15, −0.06)	−0.05 (−0.09, −0.02)	−0.16 (−0.22, −0.10)
Instrumental support Low (−1 SD)	−0.30 (−0.36, −0.24)	0.58 (0.54, 0.61)	−0.17 (−0.22, −0.12)	−0.17 (−0.21, −0.14)	−0.34 (−0.40, −0.29)
Instrumental support High (+1 SD)	−0.01 (−0.07, 0.05)	0.52 (0.48, 0.55)	−0.08 (−0.12, −0.05)	−0.005 (−0.03, 0.03)	−0.09 (−0.14, −0.04)
Social self-efficacy Low (−1 SD)	−0.14 (−0.19, −0.09)	0.48 (0.44, 0.53)	0.03 (−0.01, 0.08)	−0.07 (−0.09, −0.04)	−0.03 (−0.08, 0.02)
Social self-efficacy High (+1 SD)	0.12 (0.06, 0.16)	0.41 (0.37, 0.45)	−0.06 (−0.09, −0.02)	0.05 (0.03, 0.07)	−0.01 (−0.05, 0.03)
Hope Low (−1 SD)	−0.11 (−0.16, −0.06)	0.44 (0.40, 0.47)	0.08 (0.04, 0.12)	−0.05 (−0.07, −0.03)	0.04 (−0.01, 0.08)
Hope High (+1 SD)	0.12 (0.08, 0.17)	0.28 (0.25, 0.32)	−0.02 (−0.05, 0.01)	0.03 (0.02, 0.05)	0.01 (−0.02, 0.04)
Core self-evaluations Low (−1 SD)	0.01 (−0.04, 0.06)	0.48 (0.45, 0.52)	0.20 (0.17, 0.24)	0.005 (−0.02, 0.03)	0.21 (0.17, 0.25)
Core self-evaluations High (+1 SD)	0.24 (0.18, 0.29)	0.24 (0.21, 0.27)	0.07 (0.03, 0.11)	0.06 (0.04, 0.07)	0.13 (0.09, 0.17)
Exercise No regular exercise (low)	−0.22 (−0.29, −0.15)	0.59 (0.55, 0.63)	−0.11 (−0.16, −0.05)	−0.13 (−0.17, −0.09)	−0.24 (−0.30, −0.17)
Exercise Regular exercise (high)	−0.06 (−0.11, −0.02)	0.52 (0.49, 0.56)	−0.11 (−0.15, −0.08)	−0.03 (−0.06, −0.01)	−0.15 (−0.19, −0.10)

Continuous moderators (instrumental social support, social self-efficacy, hope, core self-evaluations) were standardized; for these, Low = 1 SD below the sample mean and High = 1 SD above. Binary moderators were coded 0/1, with Low = 0 and High = 1: cancer rehabilitation (0 = not received, 1 = received) and regular exercise (0 = no regular weekly exercise, 1 = regular weekly exercise). Path a = disability → participation; path b = participation → life satisfaction, adjusted for disability; path c′ = direct disability → life satisfaction, adjusted for participation; indirect = a × b; total = c′ + a × b. Each conditional slope is the path coefficient evaluated at the stated moderator level in the moderated mediation model for that moderator (interaction coefficients in Table III). Current employment is omitted because none of its interactions survived FDR correction. The index of moderated mediation (difference between the high and low conditional indirect associations) was 0.07 for cancer rehabilitation, 0.17 for instrumental support, 0.11 for social self-efficacy, 0.08 for hope, 0.05 for core self-evaluations, and 0.10 for exercise.

### Conditional indirect associations

Conditional indirect associations varied substantially across moderator levels (see Table IV). Instrumental social support showed the strongest buffering pattern: the conditional indirect association was β = −0.17 (95% CI −0.21 to −0.14) at low support and a non-significant β = −0.005 (95% CI −0.03 to 0.03) at high support, where it was not statistically distinguishable from zero; the conditional total association fell correspondingly, from −0.34 to −0.09. Cancer rehabilitation (β = −0.13 without vs −0.05 with rehabilitation) and regular exercise (−0.13 vs −0.03) showed the same pattern (Table IV).

Personal-resource moderators showed a distinctive pattern. For social self-efficacy, the conditional indirect association was β = −0.07 at low (−1 SD) and β = +0.05 at high (+1 SD; 95% CI 0.03 to 0.07), a sign reversal; hope showed a parallel reversal (−0.05 to +0.03; 95% CI 0.02 to 0.05), and core self-evaluations an emergence pattern (non-significant β = 0.005 at low, 95% CI −0.02 to 0.03; β = 0.06 at high, 95% CI 0.04 to 0.07). These reversals were accompanied by opposite shifts in the conditional direct association (see Table IV), so that the conditional total association of disability with life satisfaction was close to zero at both levels of social self-efficacy (−0.03, −0.01) and hope (+0.04, +0.01), and positive at both levels of core self-evaluations (+0.21, +0.13). The 3 personal resources correlated substantially with both disability (*r* = −0.33 to −0.53) and life satisfaction (*r* = 0.54 to 0.67), a configuration prone to statistical suppression (see Discussion).

### Sensitivity analyses

The covariate-adjusted model (age, gender, education, cancer rehabilitation, remission status; *N* = 2,997) confirmed the base pattern (path *a*: β = −0.20; path *b*: β = 0.52; path *c′*: β = −0.16; indirect β = −0.10, 95% CI −0.13 to −0.08; 39.2% mediated); the adjusted model explained 11.8% of variance in participation and 38.8% in life satisfaction.

Three further sensitivity analyses are summarized in Table V. With additional adjustment for cancer-specific covariates (*N* = 2,686), the disability–participation path and the indirect association attenuated but remained significant (path *a*: β = −0.10, 95% CI −0.16 to −0.04; indirect β = −0.05, 95% CI −0.07 to −0.02); fatigue was independently associated with lower participation (β = −0.25) and life satisfaction (β = −0.28). Substituting the full 12-item WCPS for the 5-item subset attenuated the disability–participation path (β = −0.04, 95% CI −0.09 to −0.003) and the indirect association (β = −0.03, 95% CI −0.05 to −0.002), which remained statistically significant but accounted for a smaller share of the unchanged total association (13.0% vs 39.8%); this pattern is consistent with the weak or positive correlations of the excluded role-specific items with disability (see Appendix S1) and is considered in the limitations. Excluding the 2 WHODAS 2.0 participation-domain items left the model essentially unchanged (path *a*: β = −0.12, 95% CI −0.17 to −0.08; indirect β = −0.07, 95% CI −0.10 to −0.05; 40.1% mediated; *N* = 2,945), indicating that the mediation is not attributable to item overlap.

**Table V:** Sensitivity analyses of the base single-mediator model: standardized coefficients with bias-corrected bootstrap 95% confidence intervals (CIs) (5,000 resamples)

Model	*N*	Path *a* β (95% CI)	Path *b* β	Path *c′* β	Indirect β (95% CI)	Proportion mediated (%)
Base model (5-item WCPS)	2,999	−0.14 (−0.18 to −0.10)	0.57	−0.12	−0.08 (−0.10 to −0.06)	39.8
Covariate-adjusted^a^	2,997	−0.20 (−0.24 to −0.16)	0.52	−0.16	−0.10 (−0.13 to −0.08)	39.2
Plus cancer-specific covariates^b^	2,686	−0.10 (−0.16 to −0.04)	0.45	−0.05	−0.05 (−0.07 to −0.02)	49.9^c^
Full 12-item WCPS as mediator	2,999	−0.04 (−0.09 to −0.003)	0.60	−0.18	−0.03 (−0.05 to −0.002)	13.0
WHODAS 2.0 excluding participation-domain items	2,945	−0.12 (−0.17 to −0.08)	0.58	−0.11	−0.07 (−0.10 to −0.05)	40.1

a Adjusted for age, gender, education, cancer rehabilitation receipt, and remission status.

b Additionally adjusted for time since first diagnosis, recurrence of the first cancer, second primary cancer, metastatic disease, treatment modality count (0–6), pain, and fatigue.

c The proportion mediated is unstable when the total association is small and should be interpreted with caution.

WCPS: Wisconsin Community Participation Scale; WHODAS 2.0: World Health Organization Disability Assessment Schedule 2.0.

## DISCUSSION

This study examined whether community participation mediates the disability–life satisfaction relationship in working-age US cancer survivors, and whether 7 contextual factors moderate this pathway differently across its segments. Participation accounted for part of the otherwise modest disability–life satisfaction association, consistent with the ICF framework’s positioning of participation as a central pathway linking impairment to subjective outcomes. The 7 contextual factors did not behave as a homogeneous category of buffers; their moderating associations were distributed unevenly across the 3 pathway segments. At high instrumental social support the conditional indirect association was not statistically distinguishable from zero, whereas at high social self-efficacy and hope it changed in sign, an unusual pattern whose interpretation is constrained by the cross-sectional design.

These results extend the small empirical literature that has tested ICF-derived mediation pathways formally. Full mediation by activity limitations has been reported in osteoarthritis (5) and a comparable pattern for community participation in fibromyalgia (6). The current findings indicate partial rather than full mediation, most plausibly because global life satisfaction in a heterogeneous survivor sample is shaped not only by participation but also by appraisal processes, including identity disruption, fear of recurrence, and financial strain (3,7). The persistent direct association is accordingly best read as an index of appraisal-related routes outside the participation pathway.

Disability accounted for only a small share of variance in participation, and yet participation accounted for a substantial share of the disability–life satisfaction association. At the same time, the absolute magnitudes are modest – disability explained 2.0% of the variance in participation in the base model – so participation is evidently shaped by many determinants beyond disability, and the pathway examined here is one contributor among several. Even modest disability-related differences in participation are associated with measurable differences in life satisfaction, consistent with qualitative accounts of survivors with chronic pain withdrawing from valued activities as symptoms accumulate (7). The clinical implication is that small absolute changes in participation may matter more than the magnitude of the underlying disability would suggest.

The buffering pattern for instrumental social support warrants attention. Consistent with the stress-buffering hypothesis of Cohen and Wills (23), emotional and instrumental support show distinct associations with symptom and distress outcomes in women with breast cancer (8). The present results locate this buffering on the disability–participation segment: the conditional path *a* fell from −0.30 at low to −0.01 at high instrumental support, and the mediated association was statistically indistinguishable from zero. Disability creates concrete obstacles to participation – transportation, dependence on others, fatigue – to which instrumental support is most directly aligned. Whether support removes these obstacles cannot be established cross-sectionally and requires longitudinal and interventional designs.

The reversal of the conditional indirect association at high social self-efficacy and hope warrants the most careful interpretation. Three explanations are tenable. The first is adaptive substitution: survivors with strong agency and pathways thinking (9) may respond to disability by adapting their modes of engagement rather than withdrawing, as documented in post-traumatic growth research with cancer survivors (10). The second is a response-style artefact: survivors high in self-efficacy and hope may rate their participation more favourably regardless of enacted level, producing an apparent positive association through shared method variance. The third is a statistical artefact of conditional estimates: when a moderator interaction is added to a small main effect, estimates at the moderator extremes can change sign through suppression or model instability without any corresponding reversal in the underlying process, particularly when that process unfolds longitudinally (11). The conditional slopes in Table IV lend some weight to this third explanation: as each resource increased, the direct association moved in the opposite direction to the indirect association, and the conditional total association remained close to zero across levels of self-efficacy and hope, so the reversal represents a redistribution between direct and indirect components rather than a net favourable association between disability and well-being; moreover, these resources correlate strongly with both disability and life satisfaction, the configuration in which suppression is most likely. The available data cannot distinguish these explanations; the reversal is a preliminary, hypothesis-generating signal that requires longitudinal replication, and ideally objective participation measures, before any clinical inference is drawn.

The unevenness of moderation across segments bears on how ICF contextual factors are modelled. Personal factors (social self-efficacy, hope, core self-evaluations) and instrumental support moderated all 3 segments, whereas cancer rehabilitation moderated the 2 segments originating in disability and exercise conversely. Factors sharing an ICF category thus acted on different combinations of segments – distinctions obscured when contextual factors are modelled only as additive predictors. Two boundaries should be kept in view: the ICF is a classification and interaction framework rather than a directional model, so these findings inform one hypothesized pathway within it rather than the framework as a whole; and body functions and structures were not directly assessed, so the ICF impairment level is represented here only indirectly, through self-reported limitations in func-tioning.

With the caution the cross-sectional design requires, the pathway-segmented pattern suggests a hypothesis of pathway-matched intervention: where participation is declining as disability accumulates, resources acting on the disability-to-participation segment (instrumental support, exercise, rehabilitation) would be prioritized; where participation is preserved but satisfaction is low, psychosocial resources acting on the appraisal-related segments (hope, social self-efficacy, core self-evaluations) would be prioritized, with exercise framed as participation preservation alongside its established role in symptom management (13). This is a hypothesis for longitudinal and interventional testing, not a recommendation for current practice, and the apparent sign reversal at high personal resources is not a basis for intervention claims.

### Limitations

Several limitations should temper interpretation. The cross-sectional design cannot establish the temporal ordering implicit in the mediation model, and cross-sectional estimates of indirect associations can be biased in magnitude and direction relative to the longitudinal process (11); the sign reversal at high social self-efficacy and hope is most vulnerable to this concern. A construct-overlap concern applies to the mediator and the outcome: the participation measure indexes satisfaction with participation rather than enacted participation, and the outcome is global life satisfaction, so their strong association may partly reflect a shared evaluative disposition; a behavioural or time-budget measure of enacted participation would not share this confound and should be incorporated in future tests of the model. Relatedly, the 5-item participation subset was selected *a priori* to remove role-specific items inapplicable to many respondents (see Appendix S1); the sensitivity analysis using the full 12-item scale shows that the indirect association, while unchanged in direction and statistical significance, is substantially smaller under the full scale (13.0% vs 39.8% of the total association), so the estimated share transmitted through participation depends on how participation is operationalized. The parallel sensitivity analysis excluding the WHODAS 2.0 participation-domain items indicates that predictor–mediator item overlap does not account for the mediation. All variables were self-reported, raising the possibility of common method variance, particularly for moderation results involving self-rated personal resources; a Harman single-factor test (first unrotated factor 37.4% of variance, below the conventional 50% threshold) is a coarse diagnostic that does not rule out method effects (14). Cancer stage and current treatment status were not collected, so residual heterogeneity across cancer trajectories cannot be excluded despite adjustment for the available cancer-specific covariates. The single binary measure of cancer rehabilitation receipt does not distinguish intensity, modality, or timing of services, so its conditional association reflects receipt of some form of rehabilitation rather than a dose–response relationship. The sample was recruited online through a vetted research participant platform and was restricted to working-age survivors who were employed or actively seeking employment, most of whom were in remission; the findings should not be extrapolated to older, retired, or non-working survivors, or to survivors in active treatment, who together constitute the majority of cancer survivors in clinical practice. Finally, confirmatory factor analyses of the multi-item scales were not estimated.

### Conclusion

In this cross-sectional national sample of working-age US cancer survivors, community participation statistically accounted for part of an overall modest association between disability and life satisfaction. Its magnitude depended substantially on how participation was operationalized: it accounted for 39.8% of the total association with the 5-item participation measure but for only 13.0% with the full 12-item scale, so the larger figure is not a robust estimate of the share transmitted through participation. The indirect association was differentially attenuated across levels of several modifiable contextual factors, most markedly instrumental social support. Apparent sign reversals at high social self-efficacy and hope require longitudinal replication before any clinical inference is drawn. These findings generate the hypothesis that survivorship care could match contextual resources to the segment of the disability-to-well-being pathway most at risk for an individual survivor; establishing whether such pathway-matched intervention improves outcomes requires longitudinal and interventional evidence. Whether a similar pattern holds for older, retired, or non-working cancer survivors remains an open question.

## Supplementary Material


