# Supplementary material for: DISABILITY, COMMUNITY PARTICIPATION, AND LIFE SATISFACTION IN WORKING-AGE CANCER SURVIVORS
Source: J Rehabil Med. 2026 Oct 29;58:46071. doi: 10.2340/jrm.v58.46071 (PMC13723030; doi:10.2340/jrm.v58.46071)
Supplement: Supplementary file 1 [file JRM-58-46071-s1.pdf]

Appendix S1

Item-level analysis supporting the five-item measure of perceived community participation

Supplement to: Disability, community participation, and life satisfaction in working-age cancer survivors (manuscript D-46071, Journal of Rehabilitation Medicine)

Rationale

The Wisconsin Community Participation Scale (WCPS) comprises 12 items, each rated on a 5-point scale comparing the respondent's actual level of participation with the level they would like (from much lower than I would like to much better than I would like). Several items presuppose occupancy of a specific life role: working part-time, working as a volunteer, performing the role of spouse or partner, parent, or son/daughter, attending school, and being an active member of a parish or church. For a respondent to whom a role does not apply — for example, a respondent without children rating the parenting item, or a full-time worker rating the part-time work item — the rating does not index disability-related restriction of desired participation. Such items can correlate weakly or even positively with disability: most notably, part-time work tends to increase when disability forces a reduction from full-time hours, so a higher part-time rating can reflect greater rather than lesser disability impact.

Before estimating the mediation models reported in the manuscript, an item-level analysis was therefore conducted to identify the subset of WCPS items that (i) apply to essentially all working-age respondents and (ii) relate to disability in the theoretically expected (negative) direction.

Method

Analyses used the final analytic sample ($N = 2{,}999$). For each WCPS item, the Pearson correlation with the 12-item World Health Organization Disability Assessment Schedule 2.0 (WHODAS 2.0) summary score was computed. Internal consistency (Cronbach's α) was computed for the full 12-item scale and for the retained 5-item subset.

Results

Table S1 reports the item-level correlations. The five retained items — full-time work, leisure and recreational activities, independent living, active social life, and low-impact physical activity — all correlated negatively with disability ($r = -0.03$ to -0.15). Of the seven excluded role-specific items, five correlated near zero or positively with disability ($r = -0.02$ to $+0.12$), with the largest positive correlations for part-time work ($r = +0.12$) and volunteer work ($r = +0.10$), consistent with the substitution dynamic described above; the spousal ($r = -0.05$) and parental ($r = -0.06$) items correlated weakly negatively but were excluded on applicability grounds (criterion i), as each presupposes a role that many respondents do not occupy. Internal consistency was $\alpha = 0.82$ for the 5-item subset and $\alpha = 0.88$ for the full 12-item scale; the two composite scores correlated $r = 0.90$.

Table S1. WCPS items, selection status, and Pearson correlation with WHODAS 2.0 disability ($N = 2{,}999$).

WCPS item	Status	r with WHODAS 2.0
Working full-time	Retained	−0.03
Working part-time	Excluded	+0.12
Working as a volunteer	Excluded	+0.10
Performing role as husband/wife/partner	Excluded	−0.05
Performing role as a parent	Excluded	−0.06
Performing role as a son or daughter	Excluded	−0.02
Going to school	Excluded	+0.06
Leisure and recreational activities	Retained	−0.13
Living independently	Retained	−0.15
Maintaining an active social life	Retained	−0.13
Active member of parish/church	Excluded	+0.01
Low-impact physical activity	Retained	−0.10

WCPS = Wisconsin Community Participation Scale; WHODAS 2.0 = World Health Organization Disability Assessment Schedule 2.0. Correlations computed in the analytic sample; positive correlations indicate higher rated participation at higher disability.

Selection decision and implications

On the basis of this analysis, and prior to estimation of the mediation models, the five-item subset was adopted as the primary measure of perceived community participation. The retained items describe participation domains applicable to essentially all working-age respondents, and their uniformly negative correlations with disability are consistent with the construct the mediation model requires (disability-related restriction of desired participation).

Because the choice of item set affects the size of the disability–participation path, the manuscript reports a sensitivity analysis substituting the full 12-item scale for the 5-item subset (manuscript Table V). Under the full scale, the indirect association remains statistically significant and in the same direction but is smaller (13.0% vs 39.8% of the total association mediated), a pattern consistent with the dilution expected when role-specific items that correlate only weakly, or positively, with disability are included. The manuscript's limitations section accordingly notes that the estimated share of the disability–life satisfaction association transmitted through participation is conditional on the participation measure used.
